# Bis[4-chloro-2-(imino­meth­yl)phenolato]copper(II) methanol disolvate

**DOI:** 10.1107/S1600536809031043

**Published:** 2009-08-19

**Authors:** Bei Qin

**Affiliations:** a Department of Pharmacy, Xi’An Medical University, Xi’an Shaanxi 721021, People’s Republic of China

## Abstract

The title compound, [Cu(C_7_H_5_ClNO)_2_]·2CH_3_OH, possesses crystallographic twofold symmetry, with the twofold axis passing through the central Cu^II^ ion. The metal centre is coordinated by two O atoms and two N atoms from two symmetry-related Schiff base ligands, forming a slightly distorted *cis*-CuN_2_O_2_ square-planar geometry. The complex mol­ecules are linked *via* the solvent methanol mol­ecules by O—H⋯O and N—H⋯O hydrogen bonds, leading to the formation of chains along the *b* axis.

## Related literature

For general background to Schiff base copper(II) complexes, see: Adsule *et al.* (2006 ▶); Erxleben & Schumacher (2001 ▶); Stewart *et al.* (1961 ▶). For related structures, see: Li & Zhang (2004 ▶); Wei *et al.* (2004 ▶).
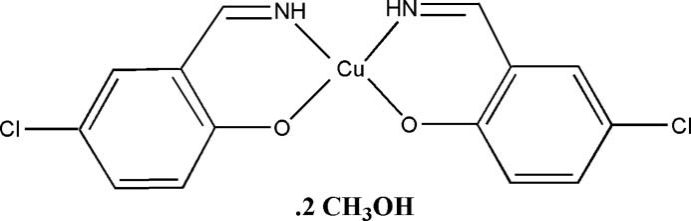


## Experimental

### 

#### Crystal data

[Cu(C_7_H_5_ClNO)_2_]·2CH_4_O
*M* *_r_* = 436.76
Monoclinic, 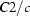

*a* = 20.603 (2) Å
*b* = 7.639 (1) Å
*c* = 14.6681 (15) Å
β = 129.376 (2)°
*V* = 1784.5 (3) Å^3^
*Z* = 4
Mo *K*α radiation
μ = 1.55 mm^−1^
*T* = 298 K
0.15 × 0.11 × 0.08 mm

#### Data collection

Bruker SMART CCD area-detector diffractometer
Absorption correction: multi-scan (*SADABS*; Sheldrick, 1996 ▶) *T*_min_ = 0.801, *T*_max_ = 0.886
4502 measured reflections
1568 independent reflections
1167 reflections with *I* > 2σ(*I*)
*R*_int_ = 0.048

#### Refinement

*R*[*F* ^2^ > 2σ(*F* ^2^)] = 0.038
*wR*(*F* ^2^) = 0.084
*S* = 1.05
1568 reflections
114 parameters
H-atom parameters constrained
Δρ_max_ = 0.38 e Å^−3^
Δρ_min_ = −0.27 e Å^−3^

### 

Data collection: *SMART* (Bruker, 2000 ▶); cell refinement: *SAINT* (Bruker, 2000 ▶); data reduction: *SAINT*; program(s) used to solve structure: *SHELXS97* (Sheldrick, 2008 ▶); program(s) used to refine structure: *SHELXL97* (Sheldrick, 2008 ▶); molecular graphics: *SHELXTL* (Sheldrick, 2008 ▶); software used to prepare material for publication: *SHELXL97*.

## Supplementary Material

Crystal structure: contains datablocks I, global. DOI: 10.1107/S1600536809031043/ci2879sup1.cif

Structure factors: contains datablocks I. DOI: 10.1107/S1600536809031043/ci2879Isup2.hkl

Additional supplementary materials:  crystallographic information; 3D view; checkCIF report

## Figures and Tables

**Table 1:** Hydrogen-bond geometry (Å, °)

*D*—H⋯*A*	*D*—H	H⋯*A*	*D*⋯*A*	*D*—H⋯*A*
O2—H2⋯O1	0.82	2.04	2.822 (3)	160
N1—H1⋯O2^i^	0.86	2.20	2.986 (4)	153

Symmetry code: (i) 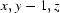.

**Fig. 1.**
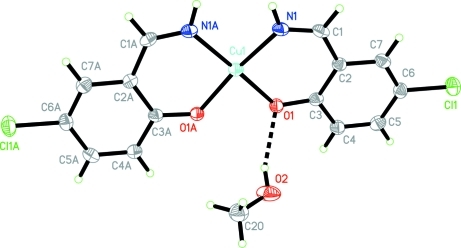
The asymmetric unit of (I), showing 30% probability displacement ellipsoids. The dashed line indicates a hydrogen bond; symmetry code: (A) 1 -x, y, 3/2 - z.

**Fig. 2.**
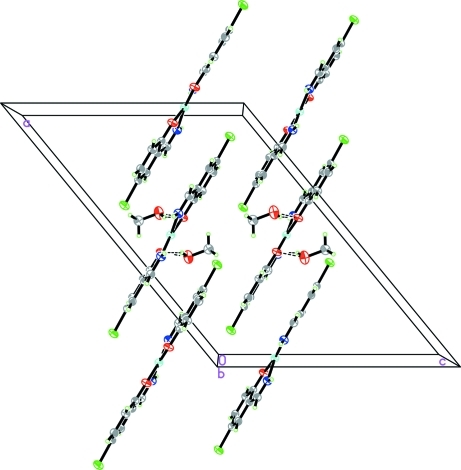
Part of the crystal packing of (I), viewed along the b axis. Hydrogen bonds are shown as dashed lines.

## supplementary crystallographic information

### Comment

The syntheses of copper(II) complexes with Schiff base have been reported for their applications in the design and construction of new magnetic materials (Erxleben & Schumache, 2001; Stewart *et al.*, 1961). Some of these complexes also inhibit the cellular proteasome activity (Adsule *et al.*, 2006). As an extension of the work on structural characterization of mononuclear copper(II) complexes, the crystal structure of the title compound is reported.

Complex (I) is a mononuclear copper(II) compound. The central Cu^II^ atom is coordinated by two O atoms and two N atoms of the two Schiff base ligands to form a slightly distorted square-planar geometry, with angles subtended at the copper(II) atoms in the range 84.48 (12)°–172.02 (10)°. The Cu—O and Cu—N bond lengths are 1.915 (2) Å and 1.939 (2) Å, respectively, which are a little longer than the corresponding value of 1.842 (3) Å and 1.837 (3) Å observed in a similar Schiff base copper(II) complex (Li & Zhang, 2004).

Intermolecular O—H···O and N—H···O hydrogen bonds involving atoms O1 and N1 from the Schiff base and O2 from the methanol (Table 1) link the molecules to form chains along the *b* axis. From Fig. 2, it can be seen that benzene rings from neighbouring complexes are parallel but the distance between their centroids is 3.852 (2) Å, which is longer than the distance (3.4 Å) between neighbouring base pairs in DNA (Wei *et al.*, 2004), indicating no π···π packing interactions.

### Experimental

All chemicals were of reagent grade and commercially available from the Beijing Chemical Reagents Company of China, and were used without further purification. 5-Chloro-2-hydroxybenzaldehyde (0.2 mmol, 31.32 mg), isopropylamine (0.2 mmol, 11.8 mg) and Cu(Ac)_2_ (0.1 mmol 18.2 mg) were dissolved in methanol (10 ml). The mixture was stirred at room temperature for 30 min and then filtered. The filtrate was allowed to stand in air for 7 d, after which time yellow block-shaped crystals of the title compound were formed by slow evaporation of the solvent.

### Refinement

H atoms attached to C and N atoms were placed in geometrically idealized positions (N-H = 0.86 Å and C-H = 0.93-0.96 Å) and constrained to ride on their parent atoms, with *U*_iso_(H) = 1.2*_U_*_eq_(C,N). H atoms attached to O atoms (water) were located in difference Fourier maps and constrained to ride on their parent atoms, with *U*_iso_(H) = 1.2*_U_*_eq_(O).

### Crystal data

**Table:**

[Cu(C_7_H_5_ClNO)_2_]·2CH_4_O	*F*(000) = 892
*M**_r_* = 436.76	*D*_x_ = 1.626 Mg m^−^^3^
Monoclinic, *C*2/*c*	Mo *K*α radiation, λ = 0.71073 Å
Hall symbol: -C 2yc	Cell parameters from 1362 reflections
*a* = 20.603 (2) Å	θ = 2.6–27.5°
*b* = 7.639 (1) Å	µ = 1.55 mm^−^^1^
*c* = 14.6681 (15) Å	*T* = 298 K
β = 129.376 (2)°	Block, yellow
*V* = 1784.5 (3) Å^3^	0.15 × 0.11 × 0.08 mm
*Z* = 4	

### Data collection

**Table:**

Bruker SMART CCD area-detector diffractometer	1568 independent reflections
Radiation source: fine-focus sealed tube	1167 reflections with *I* > 2σ(*I*)
graphite	*R*_int_ = 0.048
φ and ω scans	θ_max_ = 25.0°, θ_min_ = 2.6°
Absorption correction: multi-scan (*SADABS*; Sheldrick, 1996)	*h* = −24→20
*T*_min_ = 0.801, *T*_max_ = 0.886	*k* = −9→7
4502 measured reflections	*l* = −15→17

### Refinement

**Table:**

Refinement on *F*^2^	Primary atom site location: structure-invariant direct methods
Least-squares matrix: full	Secondary atom site location: difference Fourier map
*R*[*F*^2^ > 2σ(*F*^2^)] = 0.038	Hydrogen site location: inferred from neighbouring sites
*wR*(*F*^2^) = 0.084	H-atom parameters constrained
*S* = 1.05	*w* = 1/[σ^2^(*F*_o_^2^) + (0.0346*P*)^2^] where *P* = (*F*_o_^2^ + 2*F*_c_^2^)/3
1568 reflections	(Δ/σ)_max_ = 0.001
114 parameters	Δρ_max_ = 0.38 e Å^−^^3^
0 restraints	Δρ_min_ = −0.26 e Å^−^^3^

### Special details

**Table:**

Geometry. All e.s.d.'s (except the e.s.d. in the dihedral angle between two l.s. planes) are estimated using the full covariance matrix. The cell e.s.d.'s are taken into account individually in the estimation of e.s.d.'s in distances, angles and torsion angles; correlations between e.s.d.'s in cell parameters are only used when they are defined by crystal symmetry. An approximate (isotropic) treatment of cell e.s.d.'s is used for estimating e.s.d.'s involving l.s. planes.
Refinement. Refinement of *F*^2^ against ALL reflections. The weighted *R*-factor *wR* and goodness of fit *S* are based on *F*^2^, conventional *R*-factors *R* are based on *F*, with *F* set to zero for negative *F*^2^. The threshold expression of *F*^2^ > σ(*F*^2^) is used only for calculating *R*-factors(gt) *etc*. and is not relevant to the choice of reflections for refinement. *R*-factors based on *F*^2^ are statistically about twice as large as those based on *F*, and *R*- factors based on ALL data will be even larger.

### Fractional atomic coordinates and isotropic or equivalent isotropic displacement parameters (Å^2^)

**Table:**

	*x*	*y*	*z*	*U*_iso_*/*U*_eq_	
Cu1	0.5000	0.17304 (7)	0.7500	0.0365 (2)	
Cl1	0.11369 (5)	0.33974 (13)	0.15757 (7)	0.0592 (3)	
N1	0.41857 (15)	−0.0028 (3)	0.6418 (2)	0.0413 (7)	
H1	0.4297	−0.1088	0.6674	0.050*	
O1	0.43140 (12)	0.3587 (2)	0.64243 (18)	0.0417 (6)	
O2	0.41114 (18)	0.6573 (3)	0.7355 (2)	0.0727 (8)	
H2	0.4284	0.5693	0.7249	0.109*	
C1	0.3505 (2)	0.0205 (4)	0.5374 (3)	0.0407 (8)	
H1A	0.3177	−0.0776	0.4964	0.049*	
C2	0.32026 (18)	0.1865 (4)	0.4774 (3)	0.0342 (7)	
C3	0.36158 (18)	0.3465 (4)	0.5328 (3)	0.0343 (7)	
C4	0.32418 (18)	0.5002 (4)	0.4667 (3)	0.0414 (8)	
H4	0.3504	0.6069	0.5010	0.050*	
C5	0.25007 (19)	0.4981 (5)	0.3530 (3)	0.0439 (8)	
H5	0.2269	0.6020	0.3110	0.053*	
C6	0.21020 (18)	0.3407 (4)	0.3012 (3)	0.0408 (8)	
C7	0.24355 (19)	0.1877 (4)	0.3604 (3)	0.0411 (8)	
H7	0.2159	0.0829	0.3240	0.049*	
C20	0.4423 (2)	0.6610 (5)	0.8525 (3)	0.0618 (10)	
H20A	0.5025	0.6544	0.9048	0.093*	
H20B	0.4204	0.5632	0.8665	0.093*	
H20C	0.4254	0.7680	0.8666	0.093*	

### Atomic displacement parameters (Å^2^)

**Table:**

	*U*^11^	*U*^22^	*U*^33^	*U*^12^	*U*^13^	*U*^23^
Cu1	0.0396 (3)	0.0265 (3)	0.0406 (3)	0.000	0.0241 (3)	0.000
Cl1	0.0508 (5)	0.0672 (7)	0.0368 (5)	0.0024 (5)	0.0170 (4)	−0.0017 (4)
N1	0.0480 (16)	0.0252 (14)	0.0447 (16)	−0.0017 (13)	0.0266 (14)	−0.0018 (12)
O1	0.0385 (12)	0.0252 (12)	0.0398 (13)	−0.0011 (9)	0.0147 (11)	0.0018 (9)
O2	0.118 (2)	0.0396 (15)	0.0717 (19)	0.0145 (15)	0.0654 (18)	0.0053 (13)
C1	0.0474 (19)	0.0295 (17)	0.049 (2)	−0.0093 (15)	0.0324 (18)	−0.0109 (15)
C2	0.0352 (16)	0.0332 (17)	0.0381 (17)	0.0000 (15)	0.0251 (15)	−0.0016 (14)
C3	0.0362 (17)	0.0296 (18)	0.0398 (18)	0.0007 (14)	0.0253 (16)	0.0008 (14)
C4	0.0429 (18)	0.0338 (18)	0.0427 (19)	−0.0015 (15)	0.0248 (16)	0.0013 (15)
C5	0.050 (2)	0.0404 (19)	0.045 (2)	0.0080 (17)	0.0314 (17)	0.0103 (16)
C6	0.0379 (18)	0.050 (2)	0.0327 (17)	0.0020 (16)	0.0214 (15)	−0.0004 (15)
C7	0.0457 (19)	0.0416 (19)	0.0409 (19)	−0.0048 (17)	0.0299 (17)	−0.0087 (16)
C20	0.068 (3)	0.054 (2)	0.062 (3)	0.002 (2)	0.040 (2)	−0.001 (2)

### Geometric parameters (Å, °)

**Table:**

Cu1—O1^i^	1.9152 (19)	C2—C3	1.414 (4)
Cu1—O1	1.9152 (19)	C2—C7	1.415 (4)
Cu1—N1	1.939 (2)	C3—C4	1.402 (4)
Cu1—N1^i^	1.939 (2)	C4—C5	1.372 (4)
Cl1—C6	1.754 (3)	C4—H4	0.93
N1—C1	1.272 (4)	C5—C6	1.380 (4)
N1—H1	0.86	C5—H5	0.93
O1—C3	1.315 (3)	C6—C7	1.355 (4)
O2—C20	1.400 (4)	C7—H7	0.93
O2—H2	0.82	C20—H20A	0.96
C1—C2	1.440 (4)	C20—H20B	0.96
C1—H1A	0.93	C20—H20C	0.96
			
O1^i^—Cu1—O1	84.48 (12)	C4—C3—C2	117.4 (3)
O1^i^—Cu1—N1	172.02 (10)	C5—C4—C3	122.0 (3)
O1—Cu1—N1	92.03 (9)	C5—C4—H4	119.0
O1^i^—Cu1—N1^i^	92.03 (9)	C3—C4—H4	119.0
O1—Cu1—N1^i^	172.02 (10)	C4—C5—C6	119.6 (3)
N1—Cu1—N1^i^	92.31 (15)	C4—C5—H5	120.2
C1—N1—Cu1	127.5 (2)	C6—C5—H5	120.2
C1—N1—H1	116.2	C7—C6—C5	121.1 (3)
Cu1—N1—H1	116.2	C7—C6—Cl1	119.6 (3)
C3—O1—Cu1	128.18 (18)	C5—C6—Cl1	119.3 (2)
C20—O2—H2	109.5	C6—C7—C2	120.3 (3)
N1—C1—C2	125.3 (3)	C6—C7—H7	119.8
N1—C1—H1A	117.3	C2—C7—H7	119.8
C2—C1—H1A	117.3	O2—C20—H20A	109.5
C3—C2—C7	119.6 (3)	O2—C20—H20B	109.5
C3—C2—C1	122.7 (3)	H20A—C20—H20B	109.5
C7—C2—C1	117.6 (3)	O2—C20—H20C	109.5
O1—C3—C4	118.8 (3)	H20A—C20—H20C	109.5
O1—C3—C2	123.8 (3)	H20B—C20—H20C	109.5
			
O1—Cu1—N1—C1	4.2 (3)	C7—C2—C3—C4	−1.0 (4)
N1^i^—Cu1—N1—C1	−169.1 (3)	C1—C2—C3—C4	−177.5 (3)
O1^i^—Cu1—O1—C3	179.9 (3)	O1—C3—C4—C5	−178.1 (3)
N1—Cu1—O1—C3	−7.3 (3)	C2—C3—C4—C5	0.5 (5)
Cu1—N1—C1—C2	0.4 (5)	C3—C4—C5—C6	0.4 (5)
N1—C1—C2—C3	−4.3 (5)	C4—C5—C6—C7	−0.8 (5)
N1—C1—C2—C7	179.2 (3)	C4—C5—C6—Cl1	177.9 (2)
Cu1—O1—C3—C4	−175.7 (2)	C5—C6—C7—C2	0.3 (5)
Cu1—O1—C3—C2	5.8 (4)	Cl1—C6—C7—C2	−178.4 (2)
C7—C2—C3—O1	177.5 (3)	C3—C2—C7—C6	0.7 (5)
C1—C2—C3—O1	1.1 (5)	C1—C2—C7—C6	177.3 (3)

Symmetry codes: (i) −*x*+1, *y*, −*z*+3/2.

### Hydrogen-bond geometry (Å, °)

**Table:**

*D*—H···*A*	*D*—H	H···*A*	*D*···*A*	*D*—H···*A*
O2—H2···O1	0.82	2.04	2.822 (3)	160
N1—H1···O2^ii^	0.86	2.20	2.986 (4)	153

Symmetry codes: (ii) *x*, *y*−1, *z*.
